# Incidence of diverticulitis recurrence after sigmoid colectomy: a retrospective cohort study from a tertiary center and systematic review

**DOI:** 10.1007/s00384-023-04454-1

**Published:** 2023-06-01

**Authors:** Alexia Waser, Alexandre Balaphas, Isabelle Uhe, Christian Toso, Nicolas C. Buchs, Frédéric Ris, Jeremy Meyer

**Affiliations:** 1 Medical School, University of Geneva, Geneva, Switzerland; 2 Division of Digestive Surgery, University Hospitals of Geneva, Geneva, Switzerland

**Keywords:** Diverticulitis, Recurrence, Colectomy, Sigmoid, Sigmoidectomy

## Abstract

**Introduction:**

Our aim was to determine the incidence of diverticulitis recurrence after sigmoid colectomy for diverticular disease.

**Methods:**

Consecutive patients who benefited from sigmoid colectomy for diverticular disease from January 2007 to June 2021 were identified based on operative codes. Recurrent episodes were identified based on hospitalization codes and reviewed. Survival analysis was performed and was reported using a Kaplan–Meier curve. Follow-up was censored for last hospital visit and diverticulitis recurrence. The systematic review of the literature was performed according to the PRISMA statement. Medline, Embase, CENTRAL, and Web of Science were searched for studies reporting on the incidence of diverticulitis after sigmoid colectomy. The review was registered into PROSPERO (CRD42021237003, 25/06/2021).

**Results:**

One thousand three-hundred and fifty-six patients benefited from sigmoid colectomy. Four hundred and three were excluded, leaving 953 patients for inclusion. The mean age at time of sigmoid colectomy was 64.0 + / − 14.7 years. Four hundred and fifty-eight patients (48.1%) were males. Six hundred and twenty-two sigmoid colectomies (65.3%) were performed in the elective setting and 331 (34.7%) as emergency surgery. The mean duration of follow-up was 4.8 + / − 4.1 years. During this period, 10 patients (1.1%) developed reccurent diverticulitis. Nine of these episodes were classified as Hinchey 1a, and one as Hinchey 1b. The incidence of diverticulitis recurrence (95% CI) was as follows: at 1 year: 0.37% (0.12–1.13%), at 5 years: 1.07% (0.50–2.28%), at 10 years: 2.14% (1.07–4.25%) and at 15 years: 2.14% (1.07–4.25%). Risk factors for recurrence could not be assessed by logistic regression due to the low number of incidental cases. The systematic review of the literature identified 15 observational studies reporting on the incidence of diverticulitis recurrence after sigmoid colectomy, which ranged from 0 to 15% for a follow-up period ranging between 2 months and over 10 years.

**Conclusion:**

The incidence of diverticulitis recurrence after sigmoid colectomy is of 2.14% at 15 years, and is mostly composed of Hinchey 1a episodes. The incidences reported in the literature are heterogeneous.

**Supplementary Information:**

The online version contains supplementary material available at 10.1007/s00384-023-04454-1.

## Introduction

Colonic diverticulosis defines the presence of diverticula, which are mucosal protrusion through the *muscularis propria* of the colon. They are preferentially located in the left colon and the sigmoid colon [1]. The prevalence of diverticulosis was estimated to be as high as at 42% in an American population aged 30 to 80 years [1]. Various risk factors have been identified for the development of diverticulosis, including age, ethnicity, overweight, smoking, as well as slowed bowel movement [2]. Colonic diverticulosis remains asymptomatic in a majority of patients. However, approximately 15–20% of patients develop a complication related to diverticulosis, such as acute diverticulitis, lower gastrointestinal bleeding, colonic obstruction or chronic pain [3]. Among these complications, acute diverticulitis is the most common and affect 4–15% of patients suffering from diverticulosis during their lifetime [4]. Diverticulitis leads to significant morbidity and mortality (mortality estimated to be of 2.5 per 100,000, [5]), and represents 2.6 billion dollars per year in the USA [5].

Acute diverticulitis has been stratified by several scoring systems according to its severity [6]. The severity of the episode usually dictates its management, from medical treatment with or without antibiotics [7], to percutaneous computed tomography-guided drainage, laparoscopic lavage or Hartmann intervention [8]. In case of non-surgical management, elective sigmoid colectomy can be later performed for patients with reduced quality of life due to diverticular disease or suffering from a chronic complication of diverticular disease (such as stenosis or fistula). Moreover, emergency surgery is indicated in the event of Hinchey III or Hinchey IV diverticulitis [8]. However, sigmoid colectomy does not necessarily remove the entirety of the colon affected by diverticula. Therefore, recurrence of diverticulitis may occur after sigmoid colectomy.

Considering the importance of estimating the risk of recurrence for optimal decision making and patients’ information, we aimed at determining the incidence of recurrent diverticulitis after sigmoid colectomy.

## Materials and methods

### Original study

In this monocenter cohort study performed in a tertiary center, consecutive patients who benefited from sigmoid colectomy for diverticular disease from January 2007 to June 2021 were retrospectively identified based on operative codes. Operative reports were reviewed, and patients who benefited of sigmoid colectomy for any other indication than diverticular disease, such as cancer, were excluded. Noteworthy, indications for sigmoid colectomy for diverticular disease in our center include recurrent/chronic diverticulitis impairing quality of life and diverticular stenosis in the elective setting, and Hinchey III and Hinchey IV diverticulitis, Hinchey 1b diverticulitis not responding to non-surgical management, and diverticular bleeding not responding to endoscopic management in the emergency setting. The surgical procedure for sigmoid colectomy was performed by laparoscopy in first intent, and removed the recto-sigmoid junction. If a colorectal anastomosis was performed, the surgical procedure included mobilization of the splenic flexure to provide a tension-free anastomosis. Recurrent episodes were identified based on hospitalization codes (related to diverticulitis) and manually reviewed. A recurrent episode was defined as an episode of acute diverticulitis proven by computed tomography following sigmoid colectomy. Demographic variables at time of surgery and para-clinical variables (blood, computed tomography) at time of recurrence were collected. Survival analysis was performed and was reported using a Kaplan–Meier curve. Follow-up was censored for the last hospital visit and date of diverticulitis recurrence.

### Systematic review

The systematic review of the literature was performed according to the Preferred Reporting Items for Systematic Reviews and Meta-Analyses (PRISMA) guidelines [9] (Table S1). Medline, Embase, CENTRAL, and Web of Science were searched from inception to the 28.10.20 for studies reporting on the incidence of recurrent diverticulitis after sigmoid colectomy. The literature search strategy is reported in Table S2. Non-primary research articles (editorials, reviews, secondary analyses), duplicates, non-English literature, and studies that did not report the incidence of postoperative recurrent diverticulitis were excluded. Abstracts were considered. Two independent reviewers (AW, JM) performed the screening of eligible articles and the data extraction, using Covidence (Veritas Health Innovation, Melbourne, Australia). In case of disagreement, consensus was reached with a third reviewer (AB). Characteristics of included studies were extracted from included studies. A qualitative analysis was performed. The review was registered into the international prospective register of systematic reviews PROSPERO [10] (registration number CDR42021237003).

## Results

### Cohort study

#### Inclusion process

One thousand three hundred and fifty-six patients underwent sigmoid colectomy over the study period. Among them, 403 patients benefited from sigmoid colectomy for other indications than diverticular disease and were excluded from further analysis, leaving 953 patients for inclusion (Fig. S1).

#### Demographics of included patients

The mean age at time of sigmoid colectomy was 64.0 + / − 14.7 years. Four hundred and fifty-eight patients (48.1%) were males. Six-hundred and twenty-two sigmoid colectomies (65.3%) were performed in the elective setting and 331 (34.7%) as emergency surgery.

#### Incidence of diverticulitis recurrence after sigmoid colectomy

The mean duration of follow-up was 4.8 + / − 4.1 years. During this time period, 10 patients (1.1%) developed diverticulitis recurrence. Their mean age was 60.6 + / − 20.2 years. Six patients were females (60%) and four were males (40%). The mean white blood cells count at time of re-admission was of 11.4 + / − 8.5 G/L, and the mean C Reactiv Protein concentration was of 59.4 + / − 44.1 g/L. Five recurrent episodes were localized on the left colon above the anastomosis (colonic conduit), 3 on the right colon and 2 on the transverse colon. Nine of the recurrent episodes were classified by computed tomography as Hinchey 1a, and one was staged as Hinchey 1b (Table 1). All recurrent diverticulitis episodes were managed with antibiotics. Therefore, according to survival analysis, the incidence of recurrent diverticulitis (95% CI) was as follows: at 1 year: 0.37% (0.12–1.13%), at 5 years: 1.07% (0.50–2.28%), at 10 years: 2.14% (1.07–4.25%) and at 15 years: 2.14% (1.07–4.25%) (Fig. S2).

**Table 1:** Characteristics of patients with recurrent diverticulitis after sigmoid colectomy

			Index surgery		Recurrent episode					
Patient n°	Age, y	Sex	Setting	Anastomosis	Time to recurrence, y	WBC (G/l)	CRP (g/l)	Hinchey stage*	Localization	Hospital stay, d
1	52	Female	Elective	Yes	8.73	11	70	H1a	Right colon	5
2	78	Female	Elective	Yes	6.93	11	138	H1a	Left colon	5
3	62	Female	Elective	Yes	1.75	6	35	H1a	Right colon	4
4	85	Female	Emergency	No	0.41	7	-	H1a	Left colon	1
5	56	Male	Elective	Yes	0.98	4	7	H1a	Left colon	7
6	24	Male	Elective	Yes	5.90	19	50	H1b	Right colon	8
7	86	Female	Emergency	No	4.90	32	109	H1a	Transverse colon	-
8	74	Female	Elective	Yes	1.58	10	80	H1a	Transverse colon	5
9	43	Male	Elective	Yes	2.30	5	10	H1a	Left colon	2
10	46	Male	Elective	Yes	0.17	9	35	H1a	Left colon	7

^*^According to the modified Hinchey classification by Wasvary et al

### Systematic review of the literature

#### Inclusion process

**Fig. 1:**
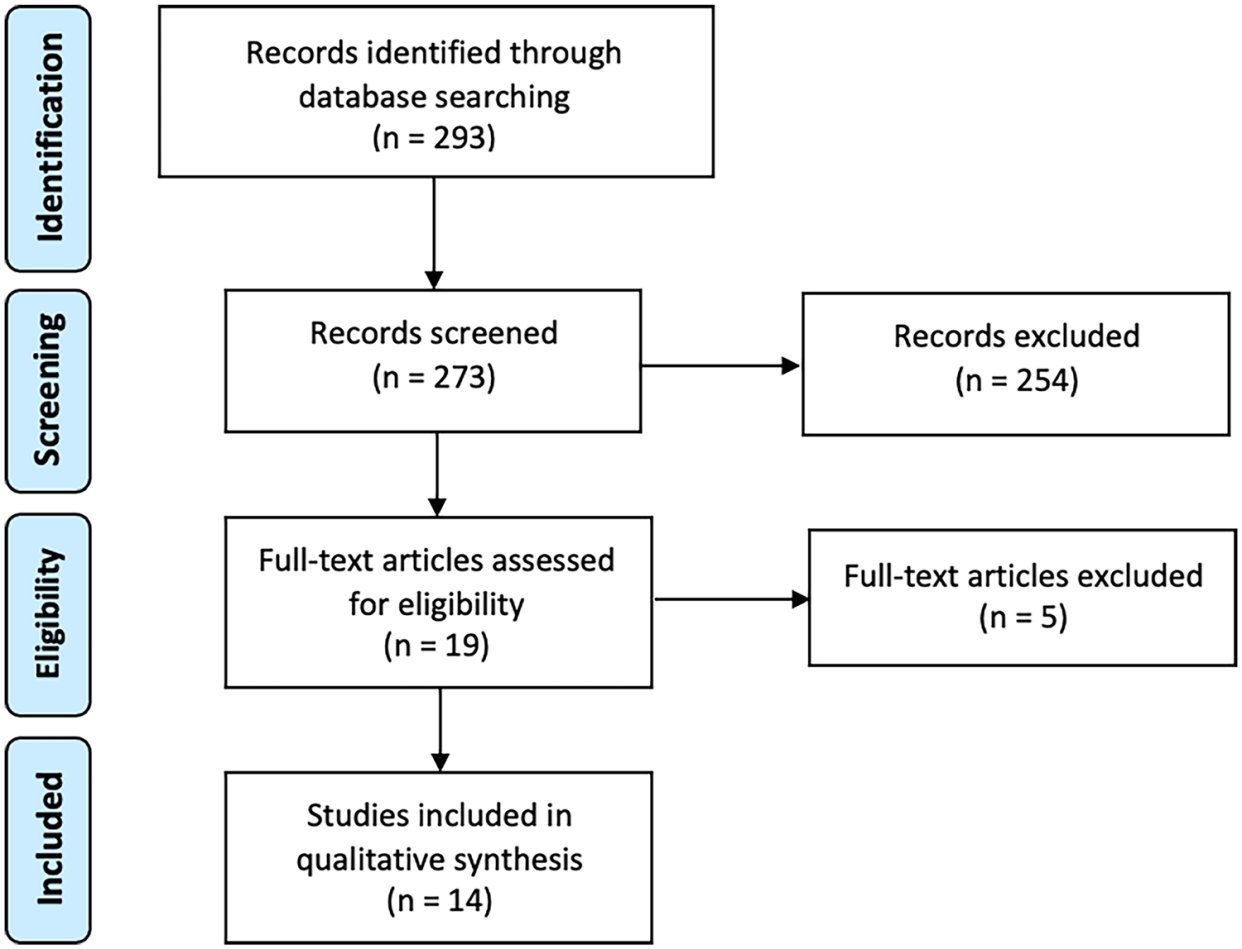
PRISMA inclusion flowchart

Two hundred ninety-three publications were identified from database screening. Twenty duplicates were removed. Of the 273 publications remaining, 254 were excluded after title and abstract screening and five after full text screening, leaving 14 studies for inclusion (Fig. 1).

#### Characteristics of included studies

Included studies totalized 4489 patients who underwent sigmoid colectomy for diverticulitis. The number of patients per study ranged between 8 [11] and 12,073 [12]. Studies were published between 2003 and 2019: two in 2003 [13, 14], one in 2004 [15], one in 2005 [16], one in 2008 [17], one in 2009 [18], one in 2011 [19], one in 2012 [20], one in 2014 [21], one in 2015 [22], one in 2016 [11], one in 2018 [23], and two in 2019 [12, 24]. Five studies were produced in the USA [12-14, 18, 24], two in France [14, 21], two in Germany [15, 19], two in Italy [20, 22], one in the UK [23], one in the Netherlands [17], one in Belgium [16], and one in Saudi Arabia [11]. The studies included 1 randomized clinical trial [23], 8 retrospective studies [12, 13, 18, 20, 22, 24,16-], 4 prospective studies [11, 15, 19, 20], and 2 case-control studies [14, 21]. Ten studies included complicated episodes of diverticulitis (11, 20, 24,15-22-] and ten studies included uncomplicated episodes of diverticulitis [12-14, 22, 24,17-]. Six studies refer to elective settings [12, 13, 16, 17, 22, 24] versus three emergency settings [17, 19, 20]. Two studies [13, 14] partly include patients from a similar cohort. Detailed characteristics of included studies are reported in Table 2.

**Table 2:** Characteristics of included studies. Number in parentheses indicate the proportion of patients with diverticulitis recurrence

Authors	Year of publication	Country	Design	Population	Patients	Recurrence	Timepoint for recurrence
Thaler et al. [13]	2003	USA	Retrospective cohort	Elective sigmoid colectomy for uncomplicated diverticulitis	236	12 (5%) confirmed clinically + CT/contrast enema	Mean follow-up 78 months (range 53–103)
Thaler et al. [14]	2003	USA/France	Case–control study	Laparoscopic vs open sigmoid colectomy for uncomplicated diverticulitis of the sigmoid	158	3 (4%) in laparoscopic group vs 7 (10%) in the open group	Mean follow-up 29 months (range 18–74)
Schwandner et al. [15]	2004	Germany	Prospective cohort	Elective laparoscopic sigmoid colectomy for acute complicated and chronic diverticular disease	396	0	Mean follow-up 20 months (range 2–72)
Laurent et al. [16]	2005	Belgium	Retrospective cohort	Laparoscopic sigmoid colectomy in patients with fistulized disease	16	0	Mean follow-up 64 months (range 7–141)
Andeweg et al. [17]	2008	The Netherlands	Retrospective cohort	Sigmoid colectomy	183	16 (8,7%)	Mean time to recurrence 38.4 months (range 6–144)
Lipof et al. [18]	2009	USA	Retrospective cohort	Laparoscopic sigmoid colectomy for diverticulitis	340	7 (2%)	Mean follow-up 42 months
Holmer et al. [19]	2011	Germany	Prospective cohort	Sigmoid colectomy for acute sigmoid diverticulitis	113 (treated surgically)	4 (3,5%)	Mean follow-up 32 months (range 12–52)
Binda et al. [20]	2012	Italy	Multicentre retrospective and prospective database analysis	Surgical treatment for acute diverticulitis	242 (treated surgically)	9 (5,8%)	Mean follow-up 128 months
Bergamaschi et al. [21]	2014	France	Case–control study	Laparoscopic vs open sigmoid colectomy for uncomplicated diverticulitis of the sigmoid	75	1 (2,7%) vs 3 (9,6%)	Follow-up 46 months
Roscio et al. [22]	2015	Italy	Retrospective cohort	Elective laparoscopic sigmoid colectomy for diverticular disease	94	0	Mean follow-up 9.6 months (range 6.9–12.3)
Ghasoup et al. [11]	2016	Saudi Arabia	Cohort	Laparoscopic sigmoid colectomy for fistulized disease	8	0	Not reported
You et al. [23]	2018	UK	Randomized clinical trial	Laparoscopic sigmoid colectomy for first episode of acute diverticulitis of the sigmoid colon complicated by extraluminal air with or without abscess vs observation	26 (treated surgically)	2 (8%)	Not reported
Choi et al. [24]	2019	USA	Retrospective cohort	Elective sigmoid colectomy for diverticulitis	361 (with long-term follow-up)	15 (4,2%)	Mean time to recurrence of 55 months (range 6–109)
Thornblade et al. [12]	2021	USA	Retrospective cohort	Elective sigmoid colectomy vs medical treatment on the second encounter for uncomplicated diverticulitis	2241 (treated surgically)	336 (15%) (at 5-year)	Follow-up 60 months

#### Incidence of diverticulitis recurrence reported by included studies

Incidence rates varied between 0 and 15% [12] for follow-up times ranging from 2 months [15] to 12 years [20]. The highest incidence was 15% over a 5-year follow-up [12]. Of 4489 pooled patients who underwent surgery and 415 had at least one episode of postoperative diverticulitis (9.2%). Recurrence was either based on clinical presentation [12], computed tomography, endoscopy, or a combination of the above [13, 14, 18, 19, 24]. The delay period required to distinguish recurrence from the previous episode varies between authors: 30 days after full recovery [19] versus 6 weeks [12].

## Discussion

According to our cohort, the incidence of diverticulitis recurrence after sigmoid colectomy for diverticular disease is of 2.14% at 15 years. These recurrences are mostly composed of uncomplicated Hinchey 1a episodes (modified classification).

The incidence of recurrence found in our population was rather low, especially when compared to the literature. For instance, one small cohort study estimated this incidence to be of 8.7% after 12 years [17]. However, this cohort was of a smaller sample size (183 patients), and diagnosis of recurrence was performed using a combination of clinical, laboratory criteria associated with compatible imaging, constituted of CT but also barium enema or colonoscopy. Therefore, the wider criteria used for diagnosing recurrence may explain the difference with our findings. Similarly, two other studies based on smaller cohorts and whose diagnostic imaging was either CT or barium enema recorded a higher incidence, respectively 5% after 6.5 years in a cohort of 236 patients [13] and 14% after 5 years in a cohort of 158 patients [14]. A larger cohort study, including 12,073 patients, of which 2241 were treated surgically, estimated the incidence of recurrence to be of 15% after 5 years for the surgical group [12]. The population included patients who received medical or surgical management (as inpatients or outpatients) for a second, third, fourth, or fifth episode of uncomplicated diverticulitis. Recurrence of diverticulitis was defined as any consultation for treatment of diverticulitis occurring more than 6 weeks after the episode; the exact diagnostic method is not mentioned. The heterogeneity in the definitions of recurrence may explain the higher incidence of recurrence than in our study. It has indeed been shown that adding an imaging method such as ultrasound or CT to the diagnosis increased the negative predictive value of diverticulitis from 0.98 to 0.99 and the positive predictive value from 0.65 to 0.95 [25]. One study showed an incidence of recurrence of 4.2% at 55 months confirmed clinically and by CT-scan [24]. However, the data were collected using emails or telephone questionnaires, which again raises the question of the exact definition of recurrence. In addition, the type of setting seems to influence the reported incidence of recurrence as shown in two studies where surgery occurred in an emergency setting for acute diverticulitis. Indeed, in these studies, the incidence of recurrence was 3.5% for a follow-up of 2.6 years [19] and 5.8% for a follow-up of 10.6 years [20]. Similarly, the surgical approach appears to influence the risk of recurrence as reported in a case–control study (2.7% recurrence in the laparoscopy group versus 9.6% for open colon resection) [21]. However, the follow-up was significantly longer in the open colon resection group (63 months) than in the laparoscopy group (46 months), which makes it difficult to draw final conclusions. The absence of diverticulitis recurrence in some studies may be explained by either the size of the cohort or the length of the follow-up [11, 15, 16, 22]. Moreover, an information bias is also possible. One randomized clinical trial recorded an incidence of recurrence of 8% [23]. However, the cohort only included patients with complicated diverticulitis. These higher incidences highlight the role of the indication as risk factor of recurrence. Nonetheless, the current literature does not support that a complicated presentation increases the risk of recurrence of diverticulitis. On the contrary, uncomplicated recurrent diverticulitis has been suggested as a likely risk factor for recurrence (the link is not established by multiple logistic regression analysis) [24]. The length of colon resection is also a postulated risk factor for recurrence. Several studies have examined this question, without finding a unanimous answer. Two case–control studies [13, 21] maintain that the level of anastomosis influences the risk of recurrence of diverticulitis after sigmoidectomy and one study [20] contradicts this hypothesis. Additionally, age and persistent postoperative pain are two risk factors of recurrence mentioned in the literature [17].

In comparison, a systematic review describes the following risk factors for recurrence of diverticulitis after non-surgical treatment. Firstly, non-modifiable factors such as young age and female gender are considered risk factors for recurrence. Secondly, it has been shown that a primary episode of acute diverticulitis with abscess formation is associated with an increased risk of recurrence, compared to uncomplicated diverticulitis. Furthermore, the size of the inflamed segment as well as the number and colonic extension of the diverticula increases the risk of recurrence. The number of previous recurrence episodes and the interval with a previous episode also plays a role. Indeed, the risk is higher in the first year after remission [26].

By comparing the risk factors for recurrence in patients treated non-surgically versus surgically, it appears that thorough selection of patients for surgical management may decrease the incidence of recurrence. Indeed, prioritizing complicated manifestations for surgery should theoretically decrease the number of recurrences of diverticulitis after sigmoidectomy. Furthermore, as the size of the inflamed segment determines the risk of recurrence in the non-surgically treated group and the level of anastomosis in the surgical group is still debated, it will be interesting to follow the literature in the future to draw conclusions.

The strengths of our study are a cohort including all stages of diverticulitis and easy access to patient follow-up data. Indeed, our center is the only public tertiary center in the region; all patients with public insurance who were operated in our center are therefore re-hospitalized in our center in case of recurrence, and the computerized system allowed us to extract follow-up data over a long period. Moreover, the review includes searches were conducted on several databases with a wide range of literature. However, it remains possible that a few patients may be treated as outpatients by their general practitioners, and may not be identified by our search in case of a recurrence. The additional limitations of our study are the absence of quality-of-life measurement after sigmoid colectomy, the fact that a few patients may have undergone sigmoid colectomy for other diverticular disease-related diagnoses, such as bleeding diverticula, and the retrospective design of the study, which limited data collection. Moreover, considering the small number of incident cases, we could not identify predictors of recurrence by logistic regression. Nevertheless, our systematic review managed to identify the following risk factors, which were consistent with a systematic review of the risk of recurrence of diverticulitis after surgery [27]: age, persistent postoperative pain (which may be a postoperative complication and not a recurrent episode), uncomplicated recurrent diverticulitis as an operative indication, preoperative diagnosis of irritable bowel syndrome, pathology result incompatible with acute diverticulitis and patient’s comorbidities. In addition, the small sample size of these studies does not allow adequate identification of risk factors by regression, and the retrospective design of these studies does not allow adequate follow-up of patients to identify possible recurrences.

## Conclusion

The risk of recurrence of diverticulitis after sigmoid colectomy is low and recurrent episodes are mostly uncomplicated. Sigmoid colectomy is therefore a good treatment option for diverticulitis. However, the indication must be weighed against the risk factors for recurrence, which should influence the proposed management.

### Supplementary Information

Below is the link to the electronic supplementary material.

Supplementary file1 (PNG 27 KB)

Supplementary file2 (PNG 371 KB)

Supplementary file3 (DOCX 20 KB)

Supplementary file4 (DOCX 14 KB)

## Data Availability

Data are available upon reasonable request.
