# Supplementary figures and images for: Incidence of diverticulitis recurrence after sigmoid colectomy: a retrospective cohort study from a tertiary center and systematic review
Source: Int J Colorectal Dis. 2023 Jun 1;38(1):157. doi: 10.1007/s00384-023-04454-1 (PMC10235134; doi:10.1007/s00384-023-04454-1)

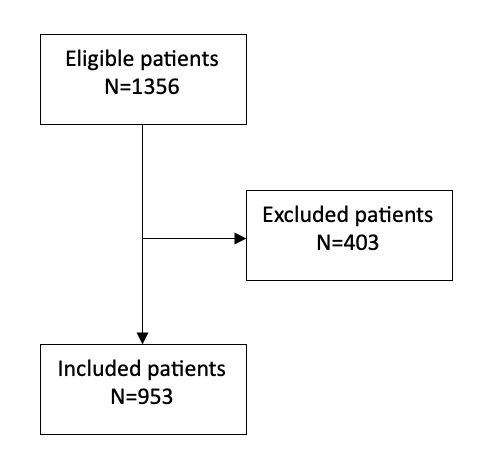

Supplement: Supplementary file 1 — Supplementary file1 (PNG 27 KB) [file 384_2023_4454_MOESM1_ESM.png]

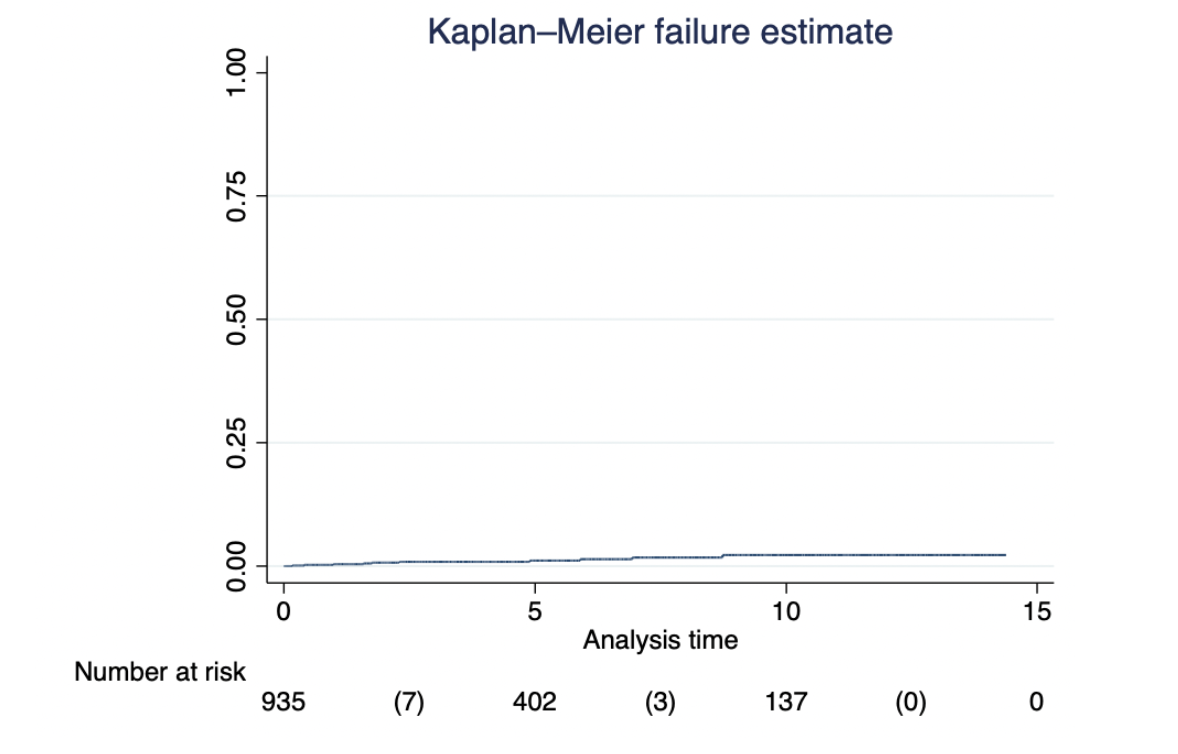

Supplement: Supplementary file 2 — Supplementary file2 (PNG 371 KB) [file 384_2023_4454_MOESM2_ESM.png]
