# Supplementary material for: Incidence of diverticulitis recurrence after sigmoid colectomy: a retrospective cohort study from a tertiary center and systematic review
Source: Int J Colorectal Dis. 2023 Jun 1;38(1):157. doi: 10.1007/s00384-023-04454-1 (PMC10235134; doi:10.1007/s00384-023-04454-1)
Supplement: Supplementary file 4 — Supplementary file4 (DOCX 14 KB) [file 384_2023_4454_MOESM4_ESM.docx]

| Database | Search build | Occurrences |
| --- | --- | --- |
| PubMed | (“diverticulitis”[MeSH] OR “diverticulitis, colonic”[MeSH] OR “colonic diverticulitis”[Title/Abstract] OR “diverticulum, colon”[MeSH] OR “colon diverticulum”[Title/Abstract] OR “colonic diverticula”[Title/Abstract] OR “colon diverticula”[Title/Abstract] OR “diverticulosis, colonic”[MeSH] OR “colonic diverticulosis”[Title/Abstract]) AND (“colectomy“[MeSH] OR “colectomies“[Title/Abstract] OR “colon resection”[Title/Abstract] OR “large bowel resection”[Title/Abstract] OR “sigmoidectomy“[Title/Abstract] OR “hartmann procedure”[Title/Abstract]) AND (“recurrence“[MeSH] OR “relapse“[Title/Abstract] OR “recurrence risk”[Title/Abstract] OR “recurrent disease”[Title/Abstract]) | 136 |
|   Embase | (‘diverticulitis’:ab,ti OR ‘colon diverticulosis’:ab,ti OR ‘colonic diverticulitis’:ab,ti OR ‘colon diverticulum’:ab,ti OR ‘colonic diverticula’:ab,ti OR ‘colon diverticula’:ab,ti OR ‘colonic diverticulosis’:ab,ti) AND (‘colectomy’:ab,ti OR ‘colectomies’:ab,ti OR ‘colon resection’:ab,ti OR ‘large bowel resection’:ab,ti OR ‘sigmoidectomy’:ab,ti OR ‘hartmann procedure’:ab,ti) AND (‘recurrence’:ab,ti OR ‘relapse’:ab,ti OR ‘recurrence risk’:ab,ti OR ‘recurrent disease’:ab,ti) | 148 |
|   Cochrane Library Wiley | (“diverticulitis”:ab,ti OR “colon diverticulosis“:ab,ti OR “colonic diverticulitis“:ab,ti OR “colon diverticulum“:ab,ti OR “colonic diverticula“:ab,ti OR “colon diverticula“:ab,ti OR “colonic diverticulosis“:ab,ti) AND  (“colectomy“:ab,ti OR “colectomies“:ab,ti OR “colon resection“:ab,ti OR “large bowel resection“:ab,ti OR “sigmoidectomy“:ab,ti OR “hartmann procedure“:ab,ti) AND (“recurrence“:ab,ti OR “relapse“:ab,ti OR “recurrence risk“:ab,ti OR “recurrent disease“:ab,ti) | 4 |
| Web of Science Core collection | TS=(“diverticulitis” OR “colon diverticulosis“ OR “colonic diverticulitis“ OR “colon diverticulum“ OR “colonic diverticula“ OR “colon diverticula“ OR “colonic diverticulosis“) AND TI=(“colectomy“ OR “colectomies“ OR “colon resection“ OR “large bowel resection“ OR “sigmoidectomy“ OR “hartmann procedure“) AND TI=(“recurrence“ OR “relapse” OR “recurrence risk“ OR “recurrent disease“) | 5 |

Table 1. Literature search strategy
